# Verification of clinically diagnosed cases during malaria elimination programme in Guizhou Province of China

**DOI:** 10.1186/1475-2875-12-130

**Published:** 2013-04-15

**Authors:** Jianhai Yin, Zhigui Xia, He Yan, Yuting Huang, Lidan Lu, Yan Geng, Ning Xiao, Jianjun Xu, Ping He, Shuisen Zhou

**Affiliations:** 1 National Institute of Parasitic Diseases, Chinese Centre for Disease Control and Prevention (NIPD, China CDC), Shanghai, 200025, China; 2 Key Laboratory of Parasite and Vector Biology, MOH, China, Shanghai, 200025, China; 3 WHO Collaborating Centre for Malaria, Schistosomiasis and Filariasis, Shanghai, 200025, China; 4 Guizhou Provincial CDC, Guiyang, 550004, China

**Keywords:** Case verification, Malaria elimination, UT-PCR, Nested PCR, Guizhou Province

## Abstract

**Background:**

China is implementing a National Malaria Elimination Programme. A high proportion of clinically diagnosed malaria cases is reported in some provinces of China. In order to understand the exact situation and make clear the nature of these patients, it is of much importance to make case verifications, particularly from the pathogenic perspective.

**Methods:**

Guizhou Province was targeted because of its high proportion of clinically diagnosed malaria cases. After random selection of around 10% of malaria cases from 1 May 2011 to 30 April 2012, reported through the national web-based case reporting system from this province, field verifications were made on 14–17 May 2012 as follows. Firstly, the reported information of each case was rechecked with the onsite case registrations and investigation forms, and an in-depth interview was conducted with each patient. Secondly, the patient’s blood smears kept by local CDC were cross-checked microscopically by a national experienced microscopist. Thirdly, two kinds of polymerase chain reaction (PCRs). including Tag-primer nested/multiplex PCR (UT-PCR) based on cytochrome oxidase gene (*cox I*) and nested PCR based on 18s rRNA gene were performed simultaneously using local CDC kept filter paper of dry blood samples to identify the *Plasmodium* spp.

**Results:**

Twelve out of 152 malaria cases were selected, including nine clinically diagnosed malaria cases, two confirmed falciparum malaria cases and one confirmed vivax malaria case. The original case documents on the site were completely in conformity with their reported data, and all the patients recalled their malaria symptoms and being cured only after consuming the corresponding anti-malarial drugs. Moreover, the re-examination results of microscopy and PCR were exactly in agreement with the original tests.

**Discussion:**

No inconsistent results were found against the reported case information in the present study and the reasons for clinically diagnosed patients remains unclear. Uniform and standardized sample collection and processing should be trained among clinicians, more sensitive and specific techniques should be explored to used in malaria diagnosis. A further study is needed in order to be more observationally focussed rather than retrospective.

## Background

As the lowest malaria burden was achieved, in order to protect the public health and respond to global malaria elimination initiative, the Chinese government launched the *Action Plan of China Malaria Elimination (2010–2020)*[1] in 2010 with an overall goal:- by 2015, local transmission of malaria should be eliminated except for partial border areas in Yunnan Province; and by 2020, malaria elimination should be achieved nationwide. In 2011, the malaria cases reported was reduced about 43.0% compared 2010, and accordingly the annual incidence was reduced to 0.0334/10,000. Furthermore, there were 56.7% vivax malaria and 40.2% falciparum malaria among the cases with laboratory confirmation [2].

The definition of malaria case in an elimination programme is very different from a control programme. In an elimination programme, it is defined as a person in whom, regardless of the presence or absence of clinical symptoms, malaria parasites have been confirmed by quality controlled laboratory diagnosis [3,4]. In the *Action Plan of China Malaria Elimination (2010–2020)*[1] as well as the *Technical Scheme of China Malaria Elimination (2011 Edition)*[5], it is the requirement that by 2012 the laboratory testing rate of malaria cases should reach 100% and the rate of laboratory confirmation should reach 75%. However, a number of clinically diagnosed or unconfirmed cases still exists. According to the national web-based case reporting system, it was noted that the proportion of clinically diagnosed malaria cases were over 25% in about one-third (10/34); Provinces/Municipalities/Autonomous Regions in China; and almost reached 90% in the key malaria-prevalent Province of Guizhou.

As every one of the malaria patients should be laboratory confirmed during elimination stage, it is necessary to make case verifications, particularly from the pathogenic perspective in order to understand the exact situation and make clear the nature of these clinically diagnosed patients.

## Methods

### Sample enrolment

Using the national web-based case reporting system, around 10% malaria cases reported from Guizhou Province during 1 May 2011 to 30 April 2012 were randomly selected into the present study, since the verifications were planned on 14–17 May 2012. All the reported case cards that had patients’ basic and diagnostic data were downloaded. All the original case registrations and investigation forms were from on site; and for each case, one slide of blood smears and one filter paper of dried blood spots kept in Guizhou provincial CDC were obtained.

### Case document review and case interview

All the reported information, including the name, gender, age, residence, date of fever onset, date and result of diagnosis, responsible health facility and doctor, etc., were compared manually with the original documents for each targeted patient. Afterwards, household interview with each patient was done in order to verify their existence, as reported.

### Ethical clearance

The study was reviewed and approved by the Ethical Committee of National Institute of Parasitic Diseases, China CDC. All patients provided informed consent before admission into the study.

### Microscopy

Both thin and thick blood smears of each Giemsa-stained slide were qualitatively evaluated and checked for malaria species by an assigned experienced microscopist from NIPD, China CDC, who was blinded to the results. The examination was performed following the WHO standard protocol under a 100× oil immersion objective [6].

### DNA extraction and PCR amplification

In Guizhou provincial CDC, two different methods for DNA extraction and polymerase chain reaction (PCR) amplification were applied simultaneously by two independent technicians: one from NIPD and one from Guizhou provincial CDC.

For the technician from Guizhou provincial CDC, a piece of 6 mm diameter dry blood sample containing around 4 μl whole blood was put into a 0.5 ml EP tube, then 400 μl PCR buffer (1×) was added, and centrifuged at 18,000×g for 2 min after 10 min standing at room temperature, then 20 μl PCR buffer (1×) added again after suspension discarded, and heated at 95°C for 5 min, centrifuged at 18,000×g for 1 min, and the suspension was the template stored at 4°C until use; a method of Tag-primer nested/multiplex PCR (UT-PCR) which can detect one parasite/μl was applied, the target gene was the cytochrome oxidase gene (*cox I*) which is located in mitochondrion, and seven primers were designed for this amplification [7].

However, for the technician from NIPD, DNA was extracted from dry blood sample using the QIAamp DNA Mini Kit (Qiagen), in accordance with the manufacturer’s instruction. Qualitative detection of *Plasmodium* spp. through nested PCR amplification based on multicopy 18s rRNA gene in a reaction of 20 μl as described elsewhere [8] with some minor adjustments. Moreover, two positive controls consisting of *Plasmodium vivax* DNA and *Plasmodium falciparum* DNA separately, and a negative control consisting of DNA extracted from malaria-negative blood and a blank control consisting of double-distilled water, were included in each PCR run. In addition, amplified regions were separated using 2% agarose gel electrophoresis and visualized following GelRed staining.

## Results

### Target province and selected samples

Guizhou Province was targeted because of its high proportion of clinically diagnosed malaria cases. From 1 May 2011 to 30 April 2012, 86.18% (131/152) reported malaria cases from this province were diagnosed as “clinical cases” according to the clinical symptoms related to malaria despite negative results of laboratory test through microscopy.

**Figure 1:**
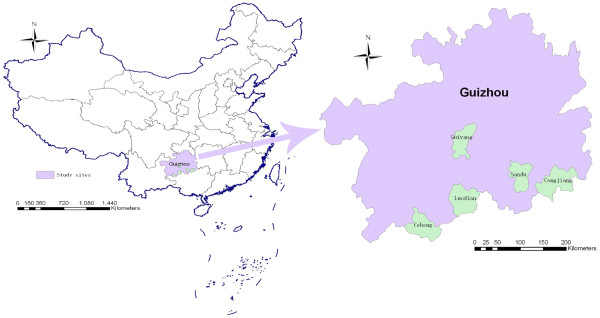
The target province and selected samples’ distribution.

Twelve out of 152 malaria cases were selected into the present study. Two confirmed imported falciparum malaria cases were from Guiyang City (code: M2, M3); nine clinically diagnosed local malaria cases were from Sandu County (code: SD1, SD2, SD6, SD8, SD11, SD19), Luodian County (code: LD5, LD12) and Ceheng County (code: CH5); and one confirmed local vivax malaria case from Congjiang County (code: CJ3) (Figure 1 and Table 1).

**Table 1:** Basic data and verification results of the study samples

**Code**	**Gender**	**Age (y)**	**Onset time**	**Original diagnosis**	***Plasmodium *spp. verified**
M2	Male	55	June 2011	Imported falciparum malaria*	*P. falciparum*
M3	Male	48	August 2011	Imported falciparum malaria*	*P. falciparum*
SD1	Male	69	May 2011	Local clinical case	Negative
SD2	Male	55	June 2011	Local clinical case	Negative
SD6	Male	16	August 2011	Local clinical case	Negative
SD8	Male	10	August 2011	Local clinical case	Negative
SD11	Female	25	August 2011	Local clinical case	Negative
SD19	Male	38	September 2011	Local clinical case	Negative
LD5	Male	53	July 2011	Local clinical case	Negative
LD12	Male	65	August 2011	Local clinical case	Negative
CJ3	Female	36	July 2011	Local vivax malaria	*P. vivax*
CH5	Female	35	April 2011	Local clinical case	Negative

* M2 attacked only one day after his return from Nigeria;

M3 attacked a week after his return from Benin.

### Case verifications

The original case documents on the site were completely in conformity with their reported data, and all the patients recalled their malaria symptoms and being cured only after consuming the corresponding anti-malarial drugs according to national guidelines [9].

Moreover, the re-examination results of microscopy and PCRs were in agreement with the original tests. The results of slides were identical to each other, tested by two different microscopists from Guizhou provincial CDC and NIPD respectively. Besides, the result of gel electrophoresis showed that *cox I* and18s rRNA genes were successfully amplified as a result of *P. falciparum* with the band around 200 bp (18s rRNA) and 620 bp (*cox I*) from two Guiyang City samples and the *P. falciparum* positive control by two methods mentioned above separately; and one sample from Congjiang County and the *P. vivax* positive control was detected as *P. vivax* around 120 bp (18s rRNA) and 220 bp (*cox I*) based on these two target genes; all the rest of the samples were negative.

## Discussion

Malaria has been a major public health problem historically in China, and China has made an impact on malaria disease burden with successful large-scale malaria control activities nationwide [10,11] and is now entering an age of malaria elimination since the *Action Plan of China Malaria Elimination (2010–2020)* was presented. In recent years, the number of malaria cases in China has been declining, with a yearly average of 46.09% [12]; most areas are free of endemic malaria, and some are in the low prevalence, this includes Guizhou, a western province bordering with Yunnan, Sichuan, Chongqing, Guangxi and Hunan Provinces/Municipalities/Autonomous Regions [13].

Generally in low transmission areas, due to the existence of asymptomatic and low parasitaemia malaria patients, prompt and effective detection/diagnosis has been a problem. Although light microscopy can characterize and quantify *Plasmodium* parasites rapidly and is the operational gold standard in control and elimination settings [14,15], the asymptomatic and micro-parasitaemia infections are beyond its detective capability [16-18]. As a result, PCR has got to be recognized as the reliable and sensitive method for malaria diagnosis, particularly to detect the submicroscopy *Plasmodium* infection, and it can identify morphologically similar species [19-21]. The methods of detecting circulating parasites by demonstrating parasite DNA through PCR assays based on 18s rRNA gene represent the overall gold standard of malaria diagnostics [22].

However in Guizhou Province, the problem is not just the asymptomatic and low-parasitaemia infection under low malaria transmission, but also that most malaria cases were reported having clinical malaria symptoms and susceptible to the anti-malarial drugs, but with no laboratory evidence of *Plasmodium* infection. In view of uncertainty in the real malaria burden of Guizhou Province, this study using retrospective design and comprehensive crosscheck approaches of case document review, case interview, microscopy and the more sensitive PCRs, attempted to understand the real malaria infections including both the asymptomatic and symptomatic, and tried to identify the differences between the results by light microscopy and PCR in such a context.

In this study, in order to ensure the accuracy of pathogenic confirmation, besides light microscopy, two kinds of PCR were used. One is nested PCR based on 18s rRNA gene, and the other is Tag-primer nested/multiplex PCR (UT-PCR) based on cytochrome oxidase gene (*cox I*). Nested PCR based on 18s rRNA gene [8] has been widely used in laboratories, clinics and field [23,24] for *Plasmodium* characterization; it demonstrated that this method was much more efficient although there are limitations. UT-PCR based on *cox I* gene in this study showed both sensitivity and specificity were higher than 98% [7], and the detection limit of either *P. falciparum* or *P. vivax* reached one parasite/μl [25]. Thus, random sampling, case document review, case interview, blood samples recheck or crosscheck through two PCRs combined with two experienced microscopists, were much more powerful for malaria detection/diagnosis theoretically.

In the present study, all the results from different methods were in good agreement with each other, and no inconsistent results were found against the reported cases information; however, the reason for clinically diagnosed patients remains unclear. It may be attributed to incorrect sampling or the clinically diagnosed patients infected with other pathogen rather than *Plasmodium* with symptoms similar to malaria, which were also susceptible to anti-malarial drugs. Although malaria diagnosis reference laboratory network has started in China since 2011, and several provincial reference laboratories have been set up in provincial CDC, respectively, it was suggested that clinicians should be trained for uniform and standardized sample collection and processing. Furthermore, it is recommended that more sensitive and specific techniques be used in malaria diagnosis, but no inconsistent results were found in this study.

## Competing interest

The authors declare that they have no competing interests.

## Authors’ contributions

JY, ZX and SZ participated in the conception of study design. Case review was carried out by JY, ZX, HY, YG and NX. Case interview and microscopy were carried out by ZX, HY, LL, JX, PH and NX. DNA extraction and PCR amplification were carried out by JY and YH. Data analysis and manuscript drafting was carried out by JY and ZX with support and contributions from LL and SZ. All authors have read and approved the final manuscript.
